# Constructal Optimization of Rectangular Microchannel Heat Sink with Porous Medium for Entropy Generation Minimization

**DOI:** 10.3390/e23111528

**Published:** 2021-11-17

**Authors:** Wenlong Li, Zhihui Xie, Kun Xi, Shaojun Xia, Yanlin Ge

**Affiliations:** 1 College of Power Engineering, Naval University of Engineering, Wuhan 430033, China; 15618509753@163.com (W.L.); xikun_91@163.com (K.X.); shaojunxia_2021@163.com (S.X.); 2 Institute of Thermal Science and Power Engineering, Wuhan Institute of Technology, Wuhan 430205, China; geyali9@hotmail.com; 3 School of Mechanical & Electrical Engineering, Wuhan Institute of Technology, Wuhan 430205, China

**Keywords:** constructal theory, entropy generation minimization, microchannel heat sink, porous medium, electronics cooling, generalized thermodynamic optimization

## Abstract

A model of rectangular microchannel heat sink (MCHS) with porous medium (PM) is developed. Aspect ratio of heat sink (HS) cell and length-width ratio of HS are optimized by numerical simulation method for entropy generation minimization (EGM) according to constructal theory. The effects of inlet Reynolds number (*Re*) of coolant, heat flux on bottom, porosity and volume proportion of PM on dimensionless entropy generation rate (DEGR) are analyzed. From the results, there are optimal aspect ratios to minimize DEGR. Given the initial condition, DEGR is 33.10% lower than its initial value after the aspect ratio is optimized. With the increase of *Re*, the optimal aspect ratio declines, and the minimum DEGR drops as well. DEGR gets larger and the optimal aspect ratio remains constant with the increasing of heat flux on bottom. For the different volume proportion of PM, the optimal aspect ratios are diverse, but the minimum DEGR almost stays unchanged. The twice minimized DEGR, which results from aspect ratio and length-width ratio optimized simultaneously, is 10.70% lower than the once minimized DEGR. For a rectangular bottom, a lower DEGR can be reached by choosing the proper direction of fluid flow.

## 1. Introduction

Electronic devices are widely used in military and civilian equipment. The components of electronics have been miniaturized and highly integrated in decades. The heat flux of electronics has increased enormously. At present, the heat flux density of some high-power electronic devices has reached 300 W/cm^2^ [1], while the local heat flux density of some high-power national defense electronic devices has reached or even exceeded 1000 W/cm^2^ [2]. In the foreseeable future, the heat flux density of electronic devices will exceed 2500 w/cm^2^ [3]. The heat must be removed in time, otherwise the devices will be destroyed because of the excessive temperature [4,5]. The thermal stress or deformation caused by temperature gradient can also dramatically decrease the reliability of devices [5]. The overheating of electronics has turned into a bottleneck in technological development. The traditional cooling techniques include air cooling, liquid cooling, heat pipe cooling, semiconductor cooling and so on. The cooling effect of air cooling is limited. The heat pipe has good heat dissipation performance, but it has the possibility of natural failure. The semiconductor cooling has the disadvantages of high cost and low efficiency. The traditional cooling techniques are unable to meet with the cooling demand of high heat flux from devices. Therefore, new methods of thermal management must be carried out for electronics. Liquid-cooled MCHSs [6,7] have lots of merits in intensive cooling capacity and are easily packaged with high density. However, due to the small volume and large flow resistance of microchannel, the pressure drop of liquid-cooled microchannel is too high, so it is of great significance to optimize it.

Heat transfer of liquid-cooled MCHS mainly consists of two processes, heat conduction and heat convection, which take place coupled at the same time. Reducing the total irreversibility of the two processes is beneficial for improving the overall thermodynamic performance. Bejan [8] firstly derived the expressions of entropy generation rate (EGR) in the processes of heat transfer and fluid flow, and proposed the principle of EGM [9,10] which enriched the optimization criterion of thermodynamic processes. A lot of researches have made important contributions to the development of EGM [11,12,13,14], and EGM has been widely used for various analyses and optimizations of processes and systems, including heat conduction [15,16], heat convection [17,18], heat exchangers [19], thermodynamic cycles [20,21], and various HSs [22,23,24,25,26,27,28,29,30,31,32,33,34,35,36,37,38,39,40,41,42,43,44,45,46,47,48,49,50,51,52,53,54,55,56,57,58,59,60,61,62,63], and so on. Moghaddam and Saedodin [22] optimized HS with square pin fins using two different algorithms for EGM, and obtained the same optimum parameters of pin fins. Al-Rashed et al. [23] established a cavity HS with circular pin fins, and obtained the numerical influences of length and number of fins on EGM in natural convection. Chauhan et al. [24] numerically compared the relative quantities of thermal EGR and frictional EGR in circular MCHS, and obtained the optimal diameter of HS with the minimum total EGR. According to the principle of bionics, Li et al. [25] established rectangular MCHS models with four kinds of fins in the light of shark-skin, and the optimal shape of fins was obtained for EGM under laminar flow conditions.

Bejan discovered the constructal law and proposed constructal theory [26]. Constructal theory has been extended by many scholars [27,28,29,30,31,32,33,34,35], and been used in heat conduction [35], heat convection [37,38,39], phase transformation [40,41], mass transfer [42], and thermodynamic cycles [43], etc. Constructal theory also has shown distinct superiority in the optimization of liquid-cooled MCHSs. Bello-Ochende et al. [44] numerically conducted the constructal design for rectangular MCHSs under the constraints of a certain volume of HS cell and a range of solid volume proportion. The optimal parameters were achieved for the lowest peak temperature when pressure drop was in the range of 10 kPa to 60 kPa. Xie et al. [45] altered the structure of MCHS by adding bifurcations in channels, which can change the flow conditions of coolant and affect the thermal performance of HS. The results indicated that there was an optimal stage of bifurcations. For triple-layered circular MCHS, constructal design was carried out by Salimpour et al. [46], and the variations of peak temperature with the diameter of every layer were obtained. Lu et al. [47] numerically analyzed the thermodynamic performance of disc HS with Y-shaped microchannel, and obtained the variation laws of peak temperature and pressure drop with branching level.

The combination of EGM and constructal theory is an important research direction in thermal design of electronics. Taking thermoeconomics and EGM into consideration, Badescu et al. [48] optimized heat exchanger according to constructal theory, and obtained the analytic solution between EGR and the parameters of smooth tube heat exchanger. With air as coolant, Chen et al. [49] acquired optimal constructs of cylindrical pin fin HS for EGM under turbulent condition, and Yang et al. [50] obtained optimal constructs with minimum operation cost defined based on EGR. Samal et al. [51] established six HS models embedded circular pipe loops with different shapes, and obtained the optimal shape and channel length of loops. Wang et al. [52] established 3-D heat dissipation model, and obtained the optimal thermal conductivity and heat generating density for EGM under turbulent conditions.

Compared with traditional MCHS, PM have two advantages simultaneously when used in MCHS. On the one hand, the specific surface area per volume of PM is larger compared with that of solid fins. On the other hand, inserting PM in the fluid region is equivalent to increase the thermal conductivity of the region. Hence, PM is extensively used in heat convection [53,54]. Bhattacharya et al. [55] analyzed the pressure drop and thermal performance of hybrid HS with PM and solid fins. Compared with the HS with PM only, the experimental results showed that heat transfer coefficient was enhanced significantly with an accessible rise of pressure drop. Hung et al. [56] numerically analyzed the heat transfer coefficient and hydraulic performance of enlarged outlet MCHS, which is fully filled with PM in channels. The results showed that enlarging the outlet can reduce the pressure drop to a certain extent, but the thermal resistance was not necessarily decreased under a low pumping power. In addition, Hung et al. [57] obtained the effect of PM shape on the performance of HS. Leng et al. [58] proposed a new MCHS whose side ribs were replaced by PM entirely. The pressure drop of the new model was nearly 50% lower compared with that of solid side ribs under the same conditions, while the peak temperature increased by 4.7%. Chen et al. [59] altered porosity distribution in the channel of MCHS, and the experimental results showed that thermal resistance was decreased by 62% compared with the conventional MCHS. Gong et al. [60] reduced the thickness of solid side ribs in the MCHSs, and replaced it with PM. The new model has exceedingly thermal and hydraulic performances compared with that of conventional solid rib model. There was an optimal dimensionless thickness of replaced ribs, which can get the best comprehensive performance. Li et al. [61] proposed a new composite HS model with solid pin fins and PM, and the thermal performance was increased by 160% compared with that of pin fin HS. Under the condition of constant wall temperature and fixed volume of PM, Ghorbani et al. [62] analyzed the thermal and hydraulic performances of MCHS partially filled with multi-layer PM, and the optimal positions of PM under different layers were obtained. In numerical assessment of HS filled with PM, Rasam et al. [63] concluded that a lower porosity or a bigger pore density of PM can evidently reduce the total EGR of HS. Wang et al. [64] numerically obtained the optimal parameters for double-layered MCHS with upper porous fins.

Bejan [65] designed the porous media with Darcy seepage flow and obtained the tree-shaped channel structure. Ordonez et al. [66] studied the asymmetric structure of a “volume-point” flow model. Wildi-Tremblay et al. [67] found that the porosity affected the pressure drop absolutely in layered porous media architecture. Lorente [68] studied the eclectrokinetic transfer from the view point of constructal design. And Lorente et al. [69] also investigated the multi-scale heterogeneous porous media. Azoumah et al. [70,71] and Zhou et al. [72] carried out the volume-point optimization for gas-solid reactors, in which heat and mass transfer simultaneously, combining EGM and constructal theory.

According to the constructal law, “For a flow system with structural evolution along the direction of the arrow of time, providing easier and easier paths for the flow flowing through its interior is the fundamental reason for the formation of its structure”. The so-called “flow system” and “flow” have broad connotations. The former includes various processes and devices in the engineering field; the latter includes fluid, heat flow, etc. For microchannel heat sink filled with porous medium, fluid passes through the porous medium and heat is also transmitted between the porous medium and the fluid. Obviously, heat sink structure has important influence on fluid flow and heat conduction.

In this paper, the open air cooling model [57] will be transformed into a closed liquid cooling microchannel model, the optimal design with entropy generation minimization for the microchannel heat sink filled with porous medium will be conducted by the constructal design method with wall peak temperature minimization in Ref. [44]. The aspect ratio of HS cell and the length-width ratio of HS will be taken as the design variables under the constraint conditions with fixed cell volume and fixed bottom area. The finite element method will be applied during the numerical simulation. The effects of inlet Reynolds number of coolant, heat flux on bottom, porosity and volume proportion of PM on DEGR will be analyzed. The results can provide theoretical guidelines for the thermal design of MCHS filled with PM.

## 2. MCHS Model

### 2.1. Geometric Model

**Figure 1:**
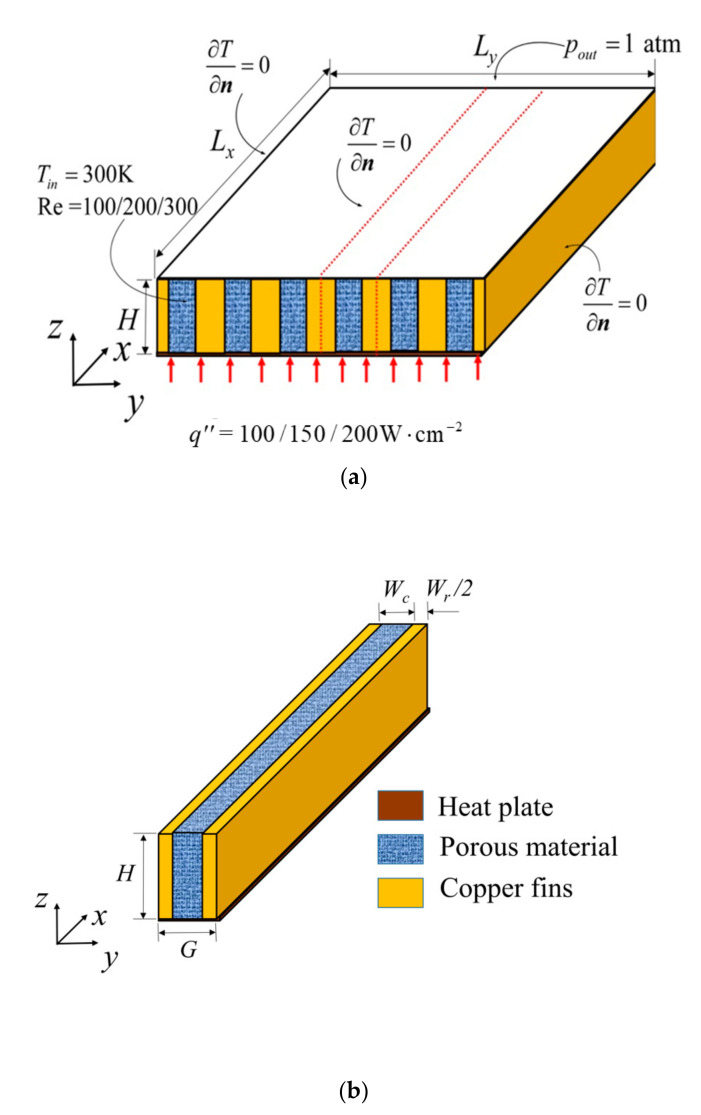
Schematic diagram of MCHS with PM, (**a**) MCHS with PM, (**b**) MCHS cell with PM.

Figure 1a shows the overall schematic diagram of HS. The length, width and height of HS are *Lx*, *Ly* and *H*, respectively. The HS is gathered by a number of cells. The cell shown in Figure 1b is selected as the unit computed, and the channel width is *Wc*. PM is fully filled in the channel. A cell consists of two solid ribs, and the thickness of each rib is $W_{r}/2$.

The volume of cell is:

(1) $$\left(W_{c}+W_{r}\right)L_{x}H=V_{c}$$

The volume of PM in cell is:

(2) $$L_{x}HW_{c}=V_{p}$$

The aspect ratio of cell is:

(3) α=H/G

The volume proportion of PM is:

(4) $$\phi_{p}=V_{p}/V_{c}$$

The total bottom area of HS is:

(5) $$S=L_{x}L_{y}$$

The length-width ratio of HS is:

(6) $$\beta =L_{x}/\;L_{y}$$

The number of cell is:

(7) $$N=L_{y}/G$$

The detail geometric parameters of HS are shown in Table 1. Under the geometric constraints of Equations (1), (2), (4) and (5), the aspect ratio of cell and the length-width ratio of HS will be optimized. Meanwhile, the effects of inlet Reynolds number of coolant, heat flux on bottom, porosity and volume proportion of PM on DEGR will be analyzed, respectively.

### 2.2. Heat Transfer Model

The following conditions are assumed about the model. The solid ribs and PM are made of isotropic copper with *k_s_* = 400 W∙m^−1^∙K^−1^. Deionized water, which is incompressible with constant properties, is selected as the coolant. The flow condition of coolant is single-phase, steady and laminar.

The governing equations of PM regions are:

(8) ∇V=0

(9) $$\frac{\rho_{f}}{\varepsilon^{2}}(V\cdot \nabla )V=-\nabla p+\frac{\mu_{f}}{\varepsilon }\nabla^{2}V-(\frac{\mu_{f}}{K}+\frac{\rho_{f}C_{F}}{\sqrt{K}}\left|V\right|)V$$

(10) $${\left(\rho c\right)}_{eff}V\nabla T=\nabla \cdot (k_{eff}\nabla T)$$

The energy equation for the solid fins:

(11) $$\nabla^{2}T=0$$

where, $V(m\cdot s^{-1})$ is the velocity vector, p(Pa) is the pressure, and μ(Pa⋅s) is the dynamic viscosity coefficient,$\;k(W\cdot m^{-1}\cdot K^{-1})$ is the thermal conductivity, T(K) is the temperature, $c(J\cdot {\mathrm{kg}}^{-1}\cdot K^{-1})$ is the constant pressure specific heat capacity. ε, $K(m^{2})$ are the porosity and permeability of PM, respectively. *C_F_* is the Forchheimer’s constant. The subscript *eff* is the effective value in the simulation. The subscripts *s* and *f* represent solid and fluid, respectively. *k_eff_* and ${\left(\rho c\right)}_{eff}$ are defined as follows:

(12) $$k_{eff}=(1-\varepsilon )k_{s}+\varepsilon k_{f}$$

(13) $${\left(\rho c\right)}_{eff}=(1-\varepsilon )\rho_{s}c_{s}+\varepsilon \rho_{f}c_{f}$$

The boundary conditions are set as follows:

Inlet temperature

(14) $$T_{in}=300K$$

Inlet Reynolds number of coolant

(15) $$Re=\rho V_{in}D_{h}/\mu_{f}$$

where, *D_h_* (m) is the hydraulic diameter, *Re* is given as 100, 200, 300, respectively.

(16) $$D_{h}=4W_{c}H/\left(2\left(W_{c}+H\right)\right)$$

Outlet pressure

(17) $$p_{out}=1^{}\mathrm{atm}$$

The interface of solid and liquid in HS

(18) $$T_{f}=T_{s}$$

(19) $$k_{f}\frac{\partial T_{f}}{\partial n}=k_{s}\frac{\partial T_{s}}{\partial n}$$

where, ***n*** is the normal direction of the interface.

A uniform heat flux $q^{\prime \prime }$ is applied on the bottom plate of HS

(20) $$q^{\prime \prime }=-k_{s}\frac{\partial T}{\partial n}$$

The other surfaces of HS are adiabatic:

(21) ∇T=0

The total pumping power of HS is:

(22) $$P=N\Delta pV_{in}W_{c}H$$

The total EGR of heat transfer process is the sum of liquid and solid parts, i.e.,:

(23) $${\dot{S}}_{g}={\dot{S}}_{g,s}+{\dot{S}}_{g,p}$$

The EGR of solid region is:

(24) $${\dot{S}}_{g,s}={\dot{S}}_{g,s,thermal}=-\int_{V}\frac{\nabla T}{T^{2}}qdV$$

where, the subscript *thermal* represents EGR caused by heat transfer.

The EGR of liquid region is:

(25) $${\dot{S}}_{g,p}={\dot{S}}_{g,p,thermal}+{\dot{S}}_{g,p,frictional}=\int_{V}(-\frac{\nabla T}{T^{2}}q+\frac{\mu }{T}\Phi^{\prime \prime \prime })dV$$

where, the subscript *frictional* represents EGR caused by fluid flow resistance. $\Phi^{\prime \prime \prime }$ is the viscous dissipation function per unit volume.

Therefore, the total EGR of the entire HS system is:

(26) $${\dot{S}}_{g}={\dot{S}}_{g,s}+{\dot{S}}_{g,p}=-\int_{V_{1}}\frac{\nabla T}{T^{2}}qdV+\int_{V_{2}}(-\frac{\nabla T}{T^{2}}q+\frac{\mu }{T}\Phi^{\prime \prime \prime })dV$$

Bejan number is defined to illustrate the contribution of EGR caused by heat transfer to the total EGR:

(27) $$Be={\dot{S}}_{g,thermal}/{\dot{S}}_{g}$$

DEGR are defined as:

(28) $$({\tilde{\dot{S}}}_{g},{\tilde{\dot{S}}}_{g,s},{\tilde{\dot{S}}}_{g,p})=\frac{({\dot{S}}_{g},{\dot{S}}_{g,s},{\dot{S}}_{g,p})T_{in}}{Q}$$

where, $Q=q^{\prime \prime }L_{x_{}}L_{y}$. The smaller ${\tilde{\dot{S}}}_{g}$ represents the less irreversibility of HS.

In this paper, COMSOL Multiphysics Server 5.5 is used for numerical simulation of the model. In the calculation, the heat sink unit is divided into tetrahedral unstructured grids. The convergence criteria of the continuity equation, momentum and energy equation are $10^{-6}$. The initial values of the model parameters are set as the following: volume ratio of porous area $\phi_{p}=0.4$, porosity ε=0.8, permeability $K=4.73\times 10^{-10}m^{2}$, coolant inlet Re=100, heat flux density at the bottom of the heat sink $q^{\prime \prime }=100W/{\mathrm{cm}}^{2}$, initial value of the aspect ratio of the end face of the heat sink α=1, overall length of the heat sink Aspect ratio β=1. Table 2 shows the heat sink entropy production rate ${\tilde{\dot{S}}}_{g}$ and relative error, calculated in this paper under different grid numbers. In order to obtain more precise results, the third grid strategy will be adopted in this paper.

## 3. Results and Discussion

### 3.1. Basic Analysis and Optimization

**Figure 2:**
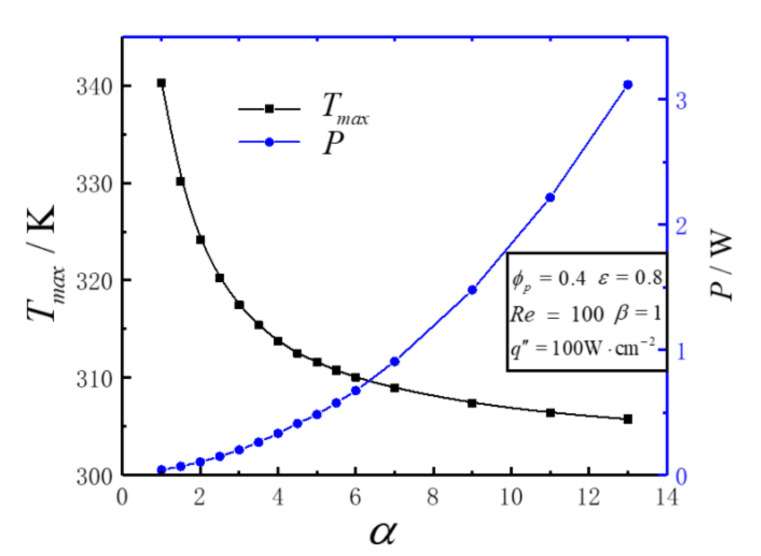
Variations of *T_max_*, *P* versus *α*.

Figure 2 illustrates the variations of maximum temperature $T_{max}$, pumping power *P* with aspect ratio *α* under the conditions of Re=100, $q^{\prime \prime }=100W\cdot {\mathrm{cm}}^{-2}$, ε=0.8, $\phi_{p}=0.4$, α=1 and β=1.

Figure 2 shows that as *α* increases, $T_{max}$ decreases gradually, but the amount of reduction becomes smaller and smaller. *P* increases with the increase of *α*. This is because increasing α increases the convective heat transfer area and strengthens the convective heat transfer of heat sink. However, on the contact surface between the porous region and the solid rib, the flow resistance also increases significantly. That is, the increase of the aspect ratio of the end face of the heat sink unit can strengthen the heat exchange and reduce the maximum temperature of the heat sink, but the price is the significant increase of the input pump power.

**Figure 3:**
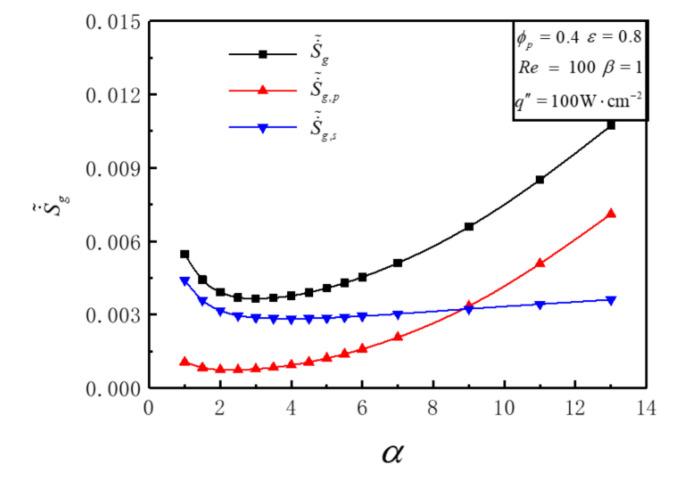
Variations of ${\tilde{\dot{S}}}_{g}$, ${\tilde{\dot{S}}}_{g,s}$, ${\tilde{\dot{S}}}_{g,p}$ versus *α*.

Figure 3 illustrates the variations of ${\tilde{\dot{S}}}_{g}$, ${\tilde{\dot{S}}}_{g,s}$, ${\tilde{\dot{S}}}_{g,p}$ with aspect ratio *α* under the conditions of Re=100, $q^{\prime \prime }=100W\cdot {\mathrm{cm}}^{-2}$, ε=0.8, $\phi_{p}=0.4$, α=1 and β=1. Figure 3 shows that there exists a critical value *α_cr_* when the value of ${\tilde{\dot{S}}}_{g,s}$ is equal to ${\tilde{\dot{S}}}_{g,p}$. ${\tilde{\dot{S}}}_{g,s}$ is bigger than ${\tilde{\dot{S}}}_{g,p}$ when *α* < *α_cr_* and vice versa. The variations of ${\tilde{\dot{S}}}_{g,s}$ and ${\tilde{\dot{S}}}_{g,p}$ with *α* are different. With the increase of *α*, ${\tilde{\dot{S}}}_{g,s}$ diminishes at first and then turns up in a very slight amount. This proves that DEGR of solid fins can not be abated continuously by increasing *α*. With the increase of *α*, ${\tilde{\dot{S}}}_{g,p}$ first reduces and then goes up, and the increasing tendency becomes larger. The variation rule of ${\tilde{\dot{S}}}_{g}$ with *α* results from the combining of ${\tilde{\dot{S}}}_{g,s}$ and ${\tilde{\dot{S}}}_{g,p}$. ${\tilde{\dot{S}}}_{g}$ decreases firstly and then increases significantly with the enlargement of *α*. There is an optimal ***α****_opt_* = 3 that makes ${\tilde{\dot{S}}}_{g}$ reach the minimum value ${\tilde{\dot{S}}}_{g,m}$ = 0.003656, and the amount of decrease is 0.001809, which is 33.10% lower than the initial value ${\tilde{\dot{S}}}_{g}$ = 0.005464. The reason is that ${\tilde{\dot{S}}}_{g,s}$ consists of heat transfer across a finite temperature difference. $T_{max}$ decreases as α increases, resulting in ${\tilde{\dot{S}}}_{g,s}$ decreasing firstly, and then keeping stable. ${\tilde{\dot{S}}}_{g,p}$ consists of heat transfer across a finite temperature difference and fluid flow with viscous effects. $T_{max}$ decreases monotonically as α increases, and EGR of heat transfer decreases firstly and then keeps stable. The channel becomes narrow when α is large, and the flow resistance increases greatly, resulting in a substantial increase in EGR by fluid flow with viscous effects.

**Figure 4:**
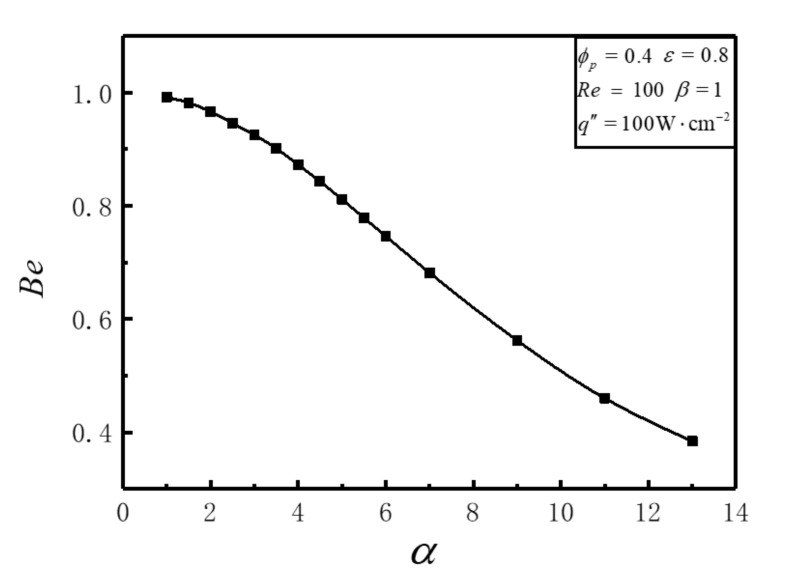
Variation of *Be* versus *α*.

Figure 4 shows that *Be* decreases continually from 0.99 to 0.38 along with the enlargement of *α* from 1 to 13. The frictional EGR is so pretty that it can be neglected when *α* = 1. But with the increase of *α*, the frictional EGR increases significantly and gradually exceeds the value of thermal EGR. This can be interpreted that increasing *α* benefits the decrease of thermal EGR under the condition with a lower *α*, but continues to increase frictional EGR. In the practical design of HS, thermal EGR and frictional EGR should both be considered. The thermal EGR should be reduced in every possible way when *Be* > 0.5, and frictional EGR should be reduced when *Be* < 0.5. The optimal aspect ratio of cell should be chosen in order to minimize the total DEGR.

### 3.2. Effects of Reynolds Number on DEGR

**Figure 5:**
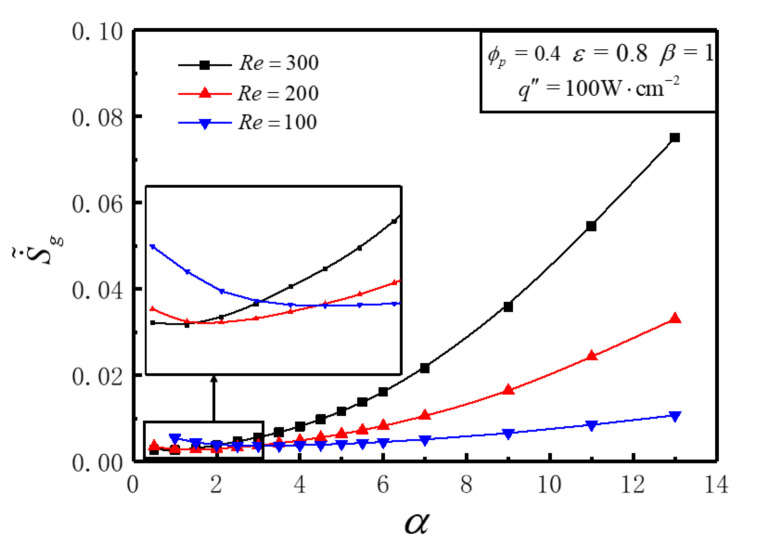
Variations of ${\tilde{\dot{S}}}_{g}$ with *α* for different *Re*.

Figure 5 illustrates the variations of ${\tilde{\dot{S}}}_{g}$ with aspect ratio *α* for different *Re* under the conditions of $q^{\prime \prime }=100W\cdot {\mathrm{cm}}^{-2}$, ε=0.8, $\phi_{p}=0.4$ and β=1. Figure 5 shows that ${\tilde{\dot{S}}}_{g}$ decreases firstly and then increases with the enlargement of *α*, and different optimal *α_opt_* can be achieved that make ${\tilde{\dot{S}}}_{g}$ reach the minimum values ${\tilde{\dot{S}}}_{g,m}$ for *Re* = 100, 200, 300, respectively. The optimal aspect ratio *α_opt_* and ${\tilde{\dot{S}}}_{g,m}$ all decrease with the increase of *Re*. For different *α*, the variation of ${\tilde{\dot{S}}}_{g}$ with *Re* is distinct. ${\tilde{\dot{S}}}_{g}$ diminishes with the enlargement of *Re* in the lower region of *α* and vice versa. There exists a certain range of *α*, which makes ${\tilde{\dot{S}}}_{g}$ decrease first and then increase with the increase of Re; i.e., there is an optimal $Re_{opt}$ to minimize ${\tilde{\dot{S}}}_{g}$. This is because the increase of *Re* diminishes thermal EGR and increases frictional EGR in the meantime. In the practical application, the relative variation of DEGRs of heat transfer and fluid friction with inlet Reynolds number of coolant should be comprehensively considered. The geometric parameter design and inlet Reynolds number for HS should be optimized simultaneously to obtain the minimum or near minimum DEGR.

Table 3 shows the optimal aspect ratio *α_opt_* of HS cell, the minimum DEGR ${\tilde{\dot{S}}}_{g,m}$, the optimal number $N_{opt}$ of cell and the hydraulic diameter *D_h_* for different *Re*. One can see that as the value of *Re* increases from 100 to 300, $\;\alpha_{opt}$ and $N_{opt}$ decrease, and the decreasing proportion of the minimum DEGR is 28.3%. The optimal *D_h_* increases, which indicates that a larger hydraulic diameter of HS cell is of advantage to minimize DEGR.

**Figure 6:**
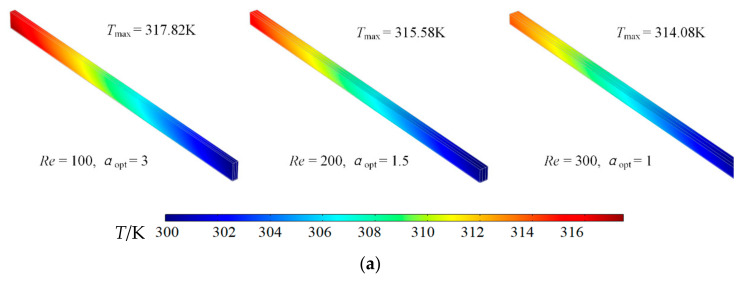

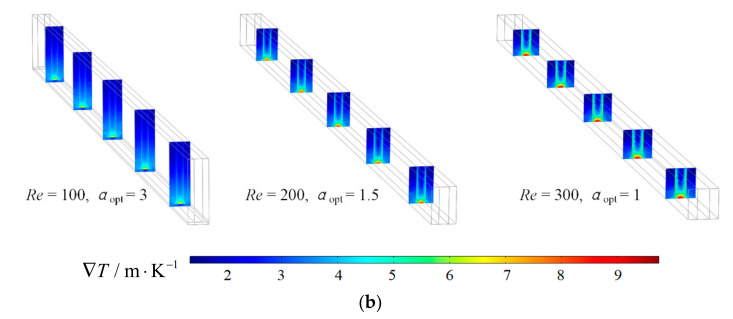
Temperature and temperature gradient profiles of optimal cell geometry for different *Re.* (**a**) Temperature profile, (**b**) Temperature gradient profile.

Figure 6 illustrates the temperature and temperature gradient profiles of optimal cell geome tries for different *Re*. Figure 6a shows that the maximum temperature of optimal cell decreases with the increase of *Re*. It indicates that increasing *Re* not only reduces the minimum DEGR, but also reduces the maximum temperature of HS. Figure 6b shows that increasing *Re* increases the maximum temperature gradient, and temperature gradient of PM region is bigger than that of solid region.

### 3.3. Effects of Heat Flux on DEGR

**Figure 7:**
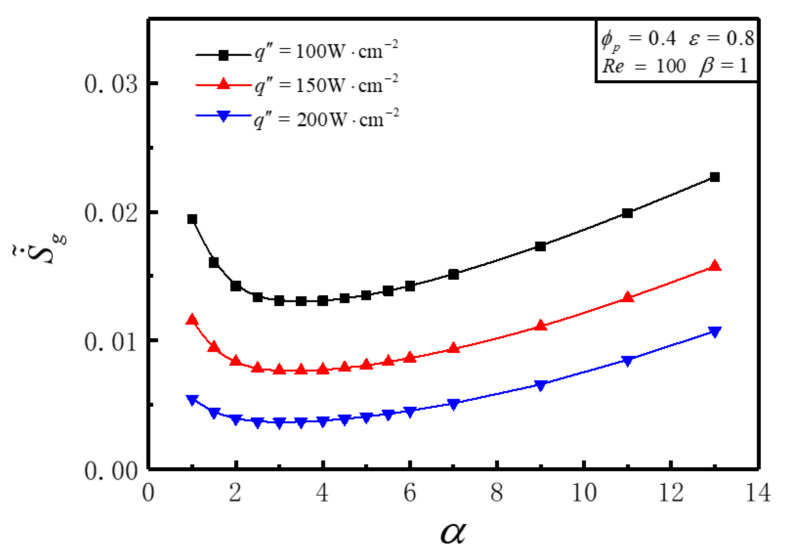
Variations of ${\tilde{\dot{S}}}_{g}$ with *α* for different $q^{\prime \prime }$.

Figure 7 illustrates the variations of ${\tilde{\dot{S}}}_{g}$ with aspect ratio *α* for different $q^{\prime \prime }$ under the conditions of Re=100, ε=0.8, $\phi_{p}=0.4$, β=1. Figure 7 shows that ${\tilde{\dot{S}}}_{g}$ increases with the increases of $q^{\prime \prime }$ for a definite *α*. ${\tilde{\dot{S}}}_{g}$ decreases firstly then increases significantly with the enlargement of *α* for a given $q^{\prime \prime }$, and an optimal *α_opt_* can be achieved that makes ${\tilde{\dot{S}}}_{g}$ get the minimum value ${\tilde{\dot{S}}}_{g,m}$. *α_opt_* stay the same value for different $q^{\prime \prime }$. This is because the increase of $q^{\prime \prime }$ increases thermal EGR directly but has little influence on frictional EGR. When $q^{\prime \prime }$ takes $100{W/\mathrm{cm}}^{2}$, $150{W/\mathrm{cm}}^{2}$, and $200{W/\mathrm{cm}}^{2}$, relative to the initial value under this condition, ${\tilde{\dot{S}}}_{g,m}$ decreases by 0.001809, 0.003898, and 0.006399, respectively, and the decrease ranges are 33.10%, 33.71%, and 32.94%, respectively. When α is given, ${\tilde{\dot{S}}}_{g}$ increases as $q^{\prime \prime }$ increases. This is because the increase of $q^{\prime \prime }$ will directly cause the increase of heat transfer entropy production rate, and the change law of flow entropy production rate with α is almost not affected by $q^{\prime \prime }$, so the law of ${\tilde{\dot{S}}}_{g}$ changing with the increase of α remains unchanged, and the value of $\alpha_{opt}$ is also constant.

### 3.4. Effects of Volume Proportion of PM on DEGR

**Figure 8:**
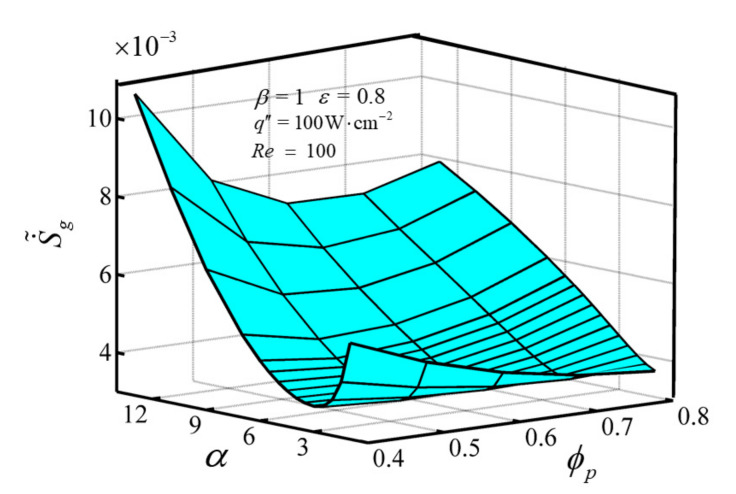
Variations of ${\tilde{\dot{S}}}_{g}$ versus *α* and $\phi_{p}$.

Figure 8 illustrates the 3-D relationship between ${\tilde{\dot{S}}}_{g}$ and aspect ratio *α*, $\phi_{p}$ under the conditions of Re=100, $q^{\prime \prime }=100W\cdot {\mathrm{cm}}^{-2}$, ε=0.8, β=1. Figure 8 shows that the variations of ${\tilde{\dot{S}}}_{g}$ with *α* are distinct for different $\phi_{p}$. There exists a critical value $\phi_{p,cr}$ that alters the changing rule of ${\tilde{\dot{S}}}_{g}$ with *α*. ${\tilde{\dot{S}}}_{g}$ has an ascent after a decline trend with the enlargement of *α* when $\phi_{p}$ < $\phi_{p,cr}$, and an optimal *α_opt_* can be achieved that makes ${\tilde{\dot{S}}}_{g}$ get the minimum value ${\tilde{\dot{S}}}_{g,m}$. ${\tilde{\dot{S}}}_{g}$ monotonically enlarges with the enlargement of *α* when $\phi_{p}$ > $\phi_{p,cr}$. The variations of ${\tilde{\dot{S}}}_{g}$ with $\phi_{p}$ are distinct for different *α*. There exists a critical value *α_cr_*, which alters the changing rule of ${\tilde{\dot{S}}}_{g}$ with $\phi_{p}$. ${\tilde{\dot{S}}}_{g}$ monotonically decreases with the increase of $\phi_{p}$ when *α* < *α_cr_*. But when *α* > *α_cr_*, optimal $\phi_{p,opt}$ can be achieved that make ${\tilde{\dot{S}}}_{g}$ get the minimum value ${\tilde{\dot{S}}}_{g,m}$ for a certain larger *α*. The maximum relative difference of the minimum DEGR is 2.2%, which illustrates that the volume proportion of PM has little effect on the minimum DEGR.

**Figure 9:**
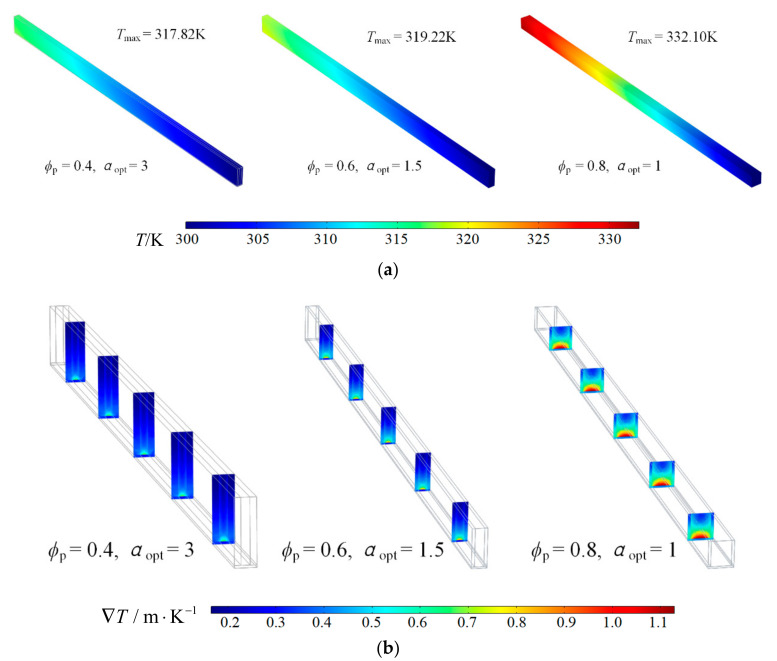
Temperature and temperature gradient profiles of optimal cell geometry for different $\phi_{p}$. (**a**) Temperature profile, (**b**) Temperature gradient profile.

Figure 9 illustrates the temperature and temperature gradient profiles of optimal cell geometry when $\phi_{p}$ are 0.4, 0.6, 0.8 respectively. Figure 9a shows that the maximum temperature of optimal cell increases with the increase of $\phi_{p}$. The reason is that a larger volume proportion of PM directly decreases the effective thermal conductivity of HS, which has negative influence on thermal conduction. Figure 9b shows that increasing volume proportion of PM increases the maximum temperature gradient.

### 3.5. Effects of Porosity on DEGR

**Figure 10:**
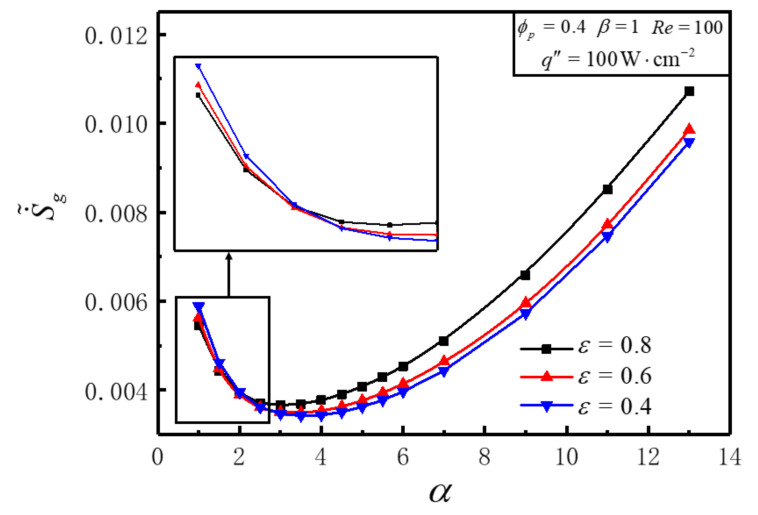
Variations of ${\tilde{\dot{S}}}_{g}$ with *α* for different *ε*.

Figure 10 illustrates the variations of ${\tilde{\dot{S}}}_{g}$ with aspect ratio *α* for different ε under the conditions of Re=100, $q^{\prime \prime }=100W\cdot {\mathrm{cm}}^{-2}$, $\phi_{p\;}=0.4$, β=1. Figure 10 shows that the variation of ${\tilde{\dot{S}}}_{g}$ decreases firstly then increases with the enlargement of *α* for any fixed ε, and an optimal *α_opt_* can be achieved that makes ${\tilde{\dot{S}}}_{g}$ get the minimum value ${\tilde{\dot{S}}}_{g,m}$. ${\tilde{\dot{S}}}_{g,m}$ enlarges and *α_opt_* goes down with ε increases. For different *α*, the variation of ${\tilde{\dot{S}}}_{g}$ with ε is distinct. ${\tilde{\dot{S}}}_{g}$ diminishes with the enlargement of ε in the lower region of *α* and vice versa. There exists a certain range of *α*, which makes ${\tilde{\dot{S}}}_{g}$ decrease first and then increases with the increase of ε, i.e., an optimal $\varepsilon_{opt}$ can be obtained for minimizing ${\tilde{\dot{S}}}_{g}$. This is because the increase of ε has positive effects on diminishing fluid friction and effective conductivity of PM region.

Table 4 shows the optimal aspect ratio *α_opt_*, the optimal number $N_{opt}$ of cell and the minimum DEGR ${\tilde{\dot{S}}}_{g,m}$ for different ε. It can be seen from Table 4 that a smaller ε makes a smaller minimum ${\tilde{\dot{S}}}_{g,m}$.

### 3.6. Effects of Length-Width Ratio on DEGR

**Figure 11:**
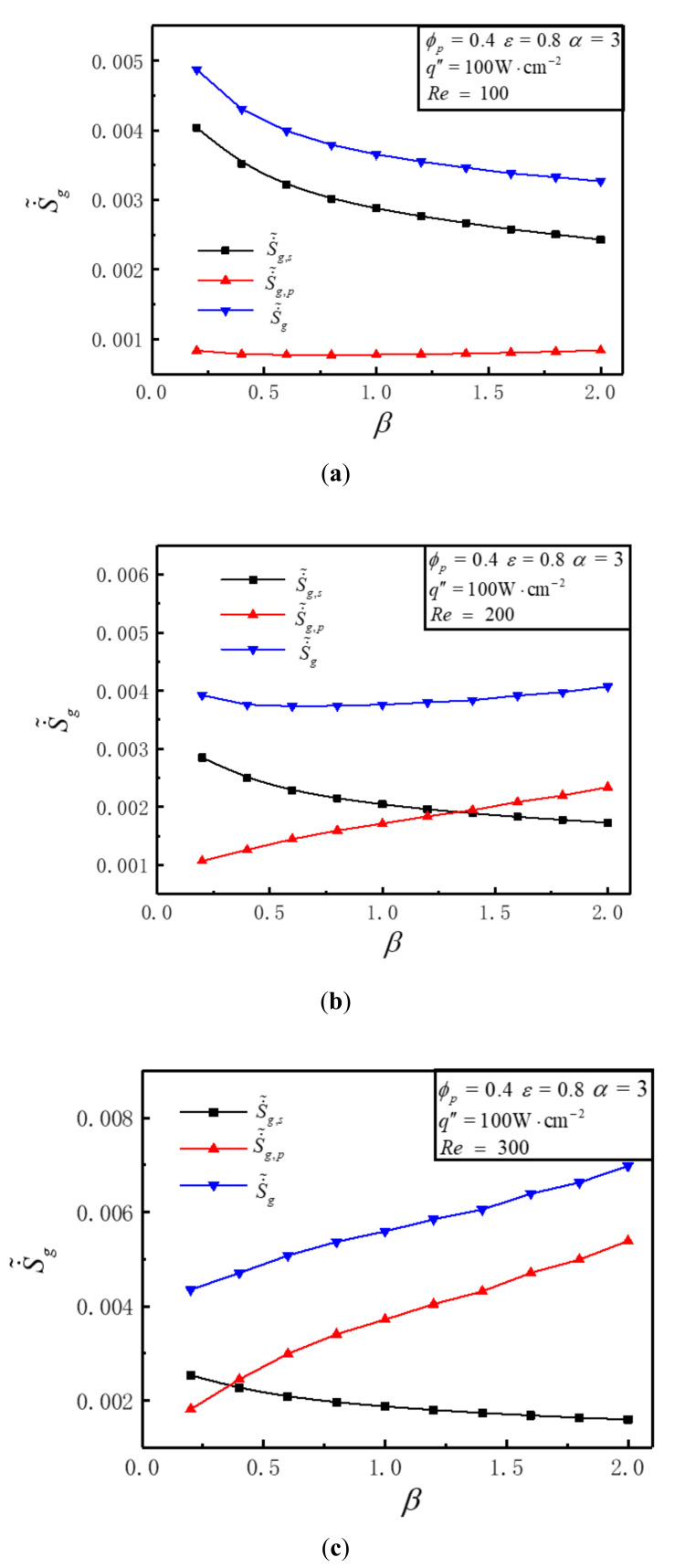
Variations of ${\tilde{\dot{S}}}_{g}$, ${\tilde{\dot{S}}}_{g,p}$, ${\tilde{\dot{S}}}_{g,s}$ with *β* for different *Re.* (**a**) *Re* = 100, (**b**) *Re* = 200, (**c**) *Re* = 300.

Figure 11 illustrates that the variations of ${\tilde{\dot{S}}}_{g}$, ${\tilde{\dot{S}}}_{g,s}$, ${\tilde{\dot{S}}}_{g,p}$ with length-width ratio *β* for different *Re* under the conditions of $q^{\prime \prime }=100W\cdot {\mathrm{cm}}^{-2}$, ε=0.8, $\phi_{p}=0.4$ and α=3. It can be seen that the variations of ${\tilde{\dot{S}}}_{g}$ are distinct for different *Re*. ${\tilde{\dot{S}}}_{g}$ decreases with the increase of *β* when *Re* = 100. ${\tilde{\dot{S}}}_{g}$ stays almost unchanged with the increase of *β* when *Re* = 200. ${\tilde{\dot{S}}}_{g}$ increases with the increase of *β* when *Re* = 300. This is because that the values of frictional EGR and thermal EGR are diverse for different *Re*. Figure 11a shows that when *Re* = 100, ${\tilde{\dot{S}}}_{g,s}$ is bigger than ${\tilde{\dot{S}}}_{g,p}$, and with the increase of *β*, ${\tilde{\dot{S}}}_{g,s}$ diminishes but ${\tilde{\dot{S}}}_{g,p}$ stays unchanged. Figure 11b shows that when *Re* = 200, the variations of ${\tilde{\dot{S}}}_{g,s}$ and ${\tilde{\dot{S}}}_{g,p}$ with the increase of *β* are absolutely opposite, i.e., ${\tilde{\dot{S}}}_{g,s}$ increases while ${\tilde{\dot{S}}}_{g,p}$ decreases. Figure 11c shows that when *Re* = 300, the amount of ${\tilde{\dot{S}}}_{g,s}$ decreased is less than that of ${\tilde{\dot{S}}}_{g,p}$ increased with the increase of *β*. The reason for the intersection of the ${\tilde{\dot{S}}}_{g,s}$ and ${\tilde{\dot{S}}}_{g,p}$ curves in Figure 11b,c is that when *Re* increases from 100 to 300, the maximum temperature of the heat sink decreases that making ${\tilde{\dot{S}}}_{g,s}$ decrease, while the flow resistance increases that making ${\tilde{\dot{S}}}_{g,p}$ increase.

### 3.7. Aspect Ratio and Length-Width Ratio Are Optimized Simultaneously for EGM

**Figure 12:**
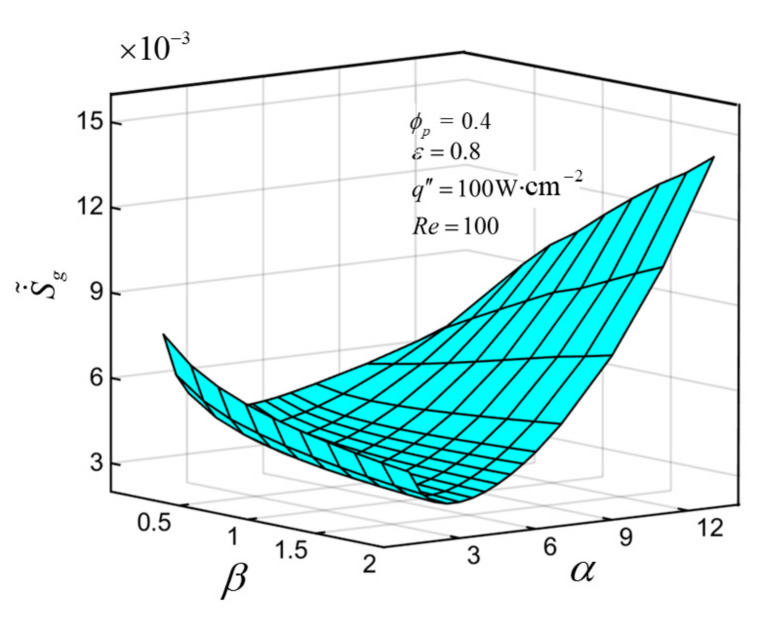
Characteristics of ${\tilde{\dot{S}}}_{g}$ versus *α* and *β*.

The aspect ratio *α* and length-width ratio *β* are optimized simultaneously for EGM. Figure 12 illustrates the variations of ${\tilde{\dot{S}}}_{g}$ versus *α* and *β* under the conditions of Re=100, $q^{\prime \prime }=100W\cdot {\mathrm{cm}}^{-2}$, ε=0.8, $\phi_{p}=0.4$. Figure 12 shows that ${\tilde{\dot{S}}}_{g}$ descends first and then ascends evidently with the enlargement of *α* for any fixed *β*, and an optimal *α_opt_* can be achieved that makes ${\tilde{\dot{S}}}_{g}$ get the minimum value ${\tilde{\dot{S}}}_{g,m}$ for a certain *β*. For different *α*, the variations of ${\tilde{\dot{S}}}_{g}$ with *β* are distinct. ${\tilde{\dot{S}}}_{g}$ diminishes with the enlargement of *β* for a certain lower *α* and vice versa. There exists a certain range of *α*, which makes ${\tilde{\dot{S}}}_{g}$ decrease first and then increase with the increase of *β*. The twice minimized DEGR ${\tilde{\dot{S}}}_{g,mm}=0.003266$ is acquired when *α* = 2.5 and *β* = 2, and ${\tilde{\dot{S}}}_{g,mm}$ is 10.70% lower than the once minimized DEGR (${\tilde{\dot{S}}}_{g,m}=0.003656$). This is because that thermal EGR will be increased by increasing *α* or *β* alone. The comprehensive influence of *α* and *β* on DEGR should be considered at the same time to obtain the minimum DEGR.

## 4. Conclusions

In this paper, a constructal optimization model for MCHS fully filled with PM is developed. The aspect ratio of HS cell and the length-width ratio of HS are optimized by numerical simulation methods combining constructal theory and EGM. The effects of inlet Reynolds number of coolant, heat flux on bottom of HS, porosity and volume proportion of PM on DEGR are analyzed, respectively. The following conclusions can be summarized.

(1) There are different aspect ratios to minimize DEGR for different Reynolds numbers. DEGR is 33.10% lower than the initial value through aspect ratio optimization.

With the increase of Reynolds number, the optimal aspect ratio and the minimum DEGR all decrease.

(2) DEGR gets larger with the increasing of heat flux, but the optimal aspect ratios remain constant. The optimal aspect ratios are diverse for different volume proportions of PM, but the maximum relative difference of the minimum DEGR is 2.2%.

(3) The twice minimized DEGR is 10.70% lower than the once minimized DEGR. For a rectangular bottom, the direction of fluid flow has a great effect on DEGR, and a lower DEGR can be reached by setting the right direction of fluid flow.

The model herein is designed for local high heat flux of high-performance computing, smart devices and high-performance CPU, and so on. In practical engineering, it can also be nested with large-scale radiators, which is the next step to design multi-structure integrated radiators with different heat dissipation load areas.

## Figures and Tables

**Table 1:** Geometrical parameters of MCHS with PM.

Geometric Parameters	Expressions	Remarks
Volume *V_c_* of the HS cell	$GL_{z}L_{x}$	Constant, 0.9 mm^3^, Geometric constraint
Bottom area *S* of the HS	$L_{x}L_{y}$	Constant, 100 mm^2^, Geometric constraint
Volume proportion $\phi_{p}$ of PM	$W_{c}/G$	Constant, Geometric constraint
Aspect ratio *α* of the cell	H/G	Independent variable
Length-width ratio *β* of the HS	$L_{x}/L_{y}$	Independent variable
Length *L_x_* of the HS	$\sqrt{\left(S/\beta \right)}$	Dependent variable
Width *L_y_* of the HS	$\beta L_{x}$	Dependent variable
Height *H* of the HS	$\sqrt{\alpha V_{c}/L_{x}}$	Dependent variable
Number *N* of cell	$L_{y}/G$	Dependent variable
Width *W_c_* of PM in a cell	$\phi_{p}\sqrt{V_{c}/L_{x}/\alpha }$	Dependent variable
Width *W_r_* of the solid fin in a cell	$(1-\phi_{p})\sqrt{V_{c}/L_{x}/\alpha }$	Dependent variable

**Table 2:** Grid independence test.

Calculation Order	Number of Units	${\tilde{\dot{S}}}_{g}$	$\left|({\tilde{\dot{S}}}_{g}^{j+1}-{\tilde{\dot{S}}}_{g}^{j})/{\tilde{\dot{S}}}_{g}^{j}\right|$
1	66,271	0.005604	—
2	182,511	0.005539	1.2%
3	356,006	0.005489	0.9%

**Table 3:** ${\tilde{\dot{S}}}_{g,m}$ and its corresponding geometric parameters for different *Re*.

*Re*	*α_opt_*	${\tilde{\dot{S}}}_{g,m}$	*N_opt_*	*D_h_*/mm
100	3	0.003656	58	0.1223
200	1.5	0.002779	41	0.1547
300	1	0.002620	33	0.1714

**Table 4:** ${\tilde{\dot{S}}}_{g,m}$ and its corresponding geometric parameters for different *ε*.

*ε*	*α_opt_*	${\tilde{\dot{S}}}_{g,m}$	*N_opt_*	*D_h_*/mm
0.4	3.5	0.003427	62	0.09681
0.6	3.5	0.003503	62	0.09681
0.8	3	0.003656	58	0.10280

## Nomenclatures

*C_F_*	Forchheimer’s constant
*c*	Constant pressure specific heat capacity, J∙kg^−1^∙K^−1^
*D_h_*	Hydraulic diameter, mm
*G*	Width of heat sink cell, mm
*K*	Permeability, m^2^
*k*	Thermal conductivity, W∙m^−1^∙K^−1^
*L_x_*	Length, mm
*L_y_*	Width, mm
*H*	Height, mm
*N*	Number of cell
***n***	Normal direction of interface
*P*	Pumping power, W
*p*	Pressure, Pa
*Q*	Total heating power, W
q	Heat flux vector, W∙cm^−2^
$q^{\prime \prime }$	Heat flux, W∙cm^−2^
*S*	Bottom area, mm^2^
${\dot{S}}_{g}$	Entropy generation rate, W∙K^−1^
*T*	Temperature, K
***V***	Velocity vector, m∙s^−1^
*V_c_*	Volume of a cell, mm^3^
*V_p_*	Volume of porous medium in a cell, mm^3^
*W_c_*	Channel width of a cell, mm
*W_r_*	Thickness of a rib, mm
**Greek Letters**	
*A*	Aspect ratio of a cell
β	Length-width ratio of heat sink
ρ	Density, kg∙m^−3^
μ	Dynamic viscosity coefficient, Pa∙s
ϕ	Volume proportion of porous medium
*ε*	Porosity
$\Phi^{\prime \prime \prime }$	Viscous dissipation function per unit volume
**Superscripts**	
~	Dimensionless
**Subscripts**	
*eff*	Effective
*f*	Fluid
*in*	Inlet
*max*	Maximum
*opt*	Optimal
*out*	Outlet
*p*	Porous medium region
*s*	Solid
**Abbreviation**	
*Be*	Bejan number
DEGR	Dimensionless entropy generation rate
EGM	Entropy generation minimization
EGR	Entropy generation rate
HS	Heat sink
MCHS	Microchannel heat sink
PM	Porous medium
*Re*	Reynolds number

## References

[1] Agostini B., Fabbri M., Park J.E., Wojtan L., Thome J.R., Michel B. (2007). State of the art of high heat flux cooling technologies. Heat Transfer Eng..

[2] Mudawar I. (2001). Assessment of high-heat-flux thermal management schemes. IEEE Trans. Compon. Packag. Technol..

[3] Ebadian M.A., Lin C.X. (2011). A review of high-heat-flux heat removal technologies. J. Heat Transfer.

[4] Erp R.V., Soleimanzadeh R., Nela L., Kampitsis G., Matioli E. (2020). Co-designing electronics with microfluidics for more sustainable cooling. Nature.

[5] Abo-Zahhad E.M., Ookawara S., Radwan A., El-Shazly A.H., ElKady M.F. (2018). Thermal and structure analyses of high concentrator solar cell under confined jet impingement cooling. Energy Convers. Manag..

[6] Dixit T., Ghosh I. (2015). Review of micro-and mini-channel heat sinks and heat exchangers for single phase fluids. Renew. Sustain. Energy Rev..

[7] Bansal N., Sehgal S.S. (2016). Fluid flow within radial micro channel heat sink: A review. Indian J. Sci. Technol..

[8] Bejan A. (1982). Entropy Generation through Heat and Fluid Flow.

[9] Bejan A. (1996). Entropy Generation Minimization.

[10] Bejan A. (1996). Entropy generation minimization: The new thermodynamics of finite-size devices and finite-time processes. J. Appl. Phys..

[11] Chen L.G., Sun F.R. (2004). Advances in Finite Time Thermodynamics: Analysis and Optimization.

[12] Bejan A. (2006). Advanced Engineering Thermodynamics.

[13] Bejan A. (2002). Fundamental of exergy analysis, entropy generation minimization and the generation of flow architecture. Int. J. Energ. Res..

[14] Bejan A. (2013). Entropy generation minimization, exergy analysis, and the constructal law. Arab. J. Sci. Eng..

[15] Li S.N., Cao B.Y. (2018). Memory behaviors of entropy production rates in heat conduction. Phys. A Stat. Mech. Its Appl..

[16] Tian X.W., Wang L.Q. (2018). Heat conduction in cylinders: Entropy generation and mathematical inequalities. Int. J. Heat Mass Transf..

[17] Avellaneda J.M., Bataille F., Toutant A., Flamant G. (2020). Variational entropy generation minimization of a channel flow: Convective heat transfer in a gas flow. Int. J. Heat Mass Transf..

[18] Berrehal H., Mabood F., Makinde O.D. (2020). Entropy-optimized radiating water/FCNTs nanofluid boundary-layer flow with convective condition. Eur. Phys. J. Plus.

[19] Sodagar A.J., Ebrahimi M.A., Farzaneh G.M., Norouzi A. (2020). Optimizing chevron plate heat exchangers based on the second law of thermodynamics and genetic algorithm. J. Therm. Anal. Calorim..

[20] Insinga A.R. (2020). The quantum friction and optimal finite-time performance of the quantum Otto cycle. Entropy.

[21] Dobre C., Grosu L., Costea M., Constantin M. (2020). Beta type Stirling engine. Schmidt and finite physical dimensions thermodynamics methods faced to experiments. Entropy.

[22] Moghaddam A.J., Saedodin S. (2013). Entropy generation minimization of pin fin heat sinks by means of metaheuristic methods. Indian J. Sci. Technol..

[23] Al-Rashed A., Kolsi L., Oztop H., Abu-Hamdeh N., Borjini M. (2017). Natural convection and entropy production in a cubic cavity heated via pin-fins heat sinks. Int. J. Heat Technol..

[24] Chauhan P.R., Kumar R., Bharj R.S. (2019). Optimization of the circular microchannel heat sink under viscous heating effect using entropy generation minimization method. Therm. Sci. Eng. Prog..

[25] Li P., Guo D.Z., Huang X.Y. (2020). Heat transfer enhancement, entropy generation and temperature uniformity analyses of shark-skin bionic modified microchannel heat sink. Int. J. Heat Mass Transf..

[26] Bejan A. (1996). Street network theory of organization in nature. J. Adv. Transp..

[27] Bejan A. (1997). Constructal-theory network of conducting paths for cooling a heat generating volume. Int. J. Heat Mass Tranf..

[28] Chen L.G. (2012). Progress in study on constructal theory and its applications. Sci. China Technol. Sci..

[29] Bejan A. (2000). Shape and Structure, from Engineering to Nature.

[30] Bejan A., Lorente S. (2008). Design with Constructal Theory.

[31] Rocha L.A.O., Lorente S., Bejan A. (2013). Constructal Law and the Unifying Principle of Design.

[32] Chen L.G., Feng H.J. (2016). Multi-Objective Constructal Optimization for Flow and Heat and Mass Transfer Processes.

[33] Lorente S., Bejan A. (2019). Current trends in constructal law and evolutionary design. Heat Transfer Asian Res..

[34] Chen L.G., Feng H.J., Xie Z.H., Sun F.R. (2019). Progress of constructal theory in China over the past decade. Int. J. Heat Mass Transf..

[35] Chen L.G., Yang A.B., Feng H.J., Ge Y.L., Xia S.J. (2020). Constructal design progress for eight types of heat sinks. Sci. China Technol. Sci..

[36] Chen L.G., You J., Feng H.J., Xie Z.H. (2019). Constructal optimization for “disc-point” heat conduction with nonuniform heat generating. Int. J. Heat Mass Transf..

[37] Shi H.N., Xie Z.H., Chen L.G., Sun F.R. (2018). Constructal optimization for line-to-line vascular based on entropy generation minimization principle. Int. J. Heat Mass Transf..

[38] Pedroti V.A., Escobar C.C., dos Santos E.D., Souza J.A. (2020). Thermal analysis of tubular arrangements submitted to external flow using constructal theory. Int. Comm. Heat Mass Transf..

[39] Ariyo D.O., Bello-Ochende T. (2020). Constructal design of subcooled microchannel heat exchangers. Int. J. Heat Mass Transf..

[40] Cai C.G., Feng H.J., Chen L.G., Wu Z.X. (2019). Constructal design of a shell-and-tube evaporator with ammonia-water working fluid. Int. J. Heat Mass Transf..

[41] Feng H.J., Xie Z.J., Chen L.G., Wu Z.X., Xia S.J. (2020). Constructal design for supercharged boiler superheater. Energy.

[42] Feng H.J., Chen L.G., Xie Z.H., Sun F.R. (2015). “Disc-point” heat and mass transfer constructal optimization for solid-gas reactors based on entropy generation minimization. Energy.

[43] Wu Z.X., Feng H.J., Chen L.G., Tang W. (2020). Constructal thermodynamic optimization for ocean thermal energy conversion system with dual-pressure organic Rankine cycle. Energy Convers. Manag..

[44] Bello-Ochende T., Meyer J.P., Ighalo F.U. (2010). Combined numerical optimization and constructal theory for the design of microchannel heat sinks. Numer. Heat Transfer Part A.

[45] Xie G.N., Zhang F.L., Sundén B., Zhang W.H. (2014). Constructal design and thermal analysis of microchannel heat sinks with multistage bifurcations in single-phase liquid flow. Appl. Therm. Eng..

[46] Salimpour M.R., Al-Sammarraie A.T., Forouzandeh A., Farzaneh M. (2019). Constructal design of circular multilayer microchannel heat sinks. J. Therm. Sci. Eng. Appl..

[47] Lu Z.H., Zhang K., Liu J.X., Li F. (2020). Effect of branching level on the performance of constructal theory based Y-shaped liquid cooling heat sink. Appl. Therm. Eng..

[48] Badescu V., Baracu T., Avram R., Grigore R., Patrascu M. (2018). On the design and optimization of constructal networks of heat exchangers by considering entropy generation minimization and thermoeconomics. Proc. Rom. Acad..

[49] Chen L.G., Yang A.B., Xie Z.H., Sun F.R. (2017). Constructal entropy generation rate minimization for cylindrical pin-fin heat sinks. Int. J. Therm. Sci..

[50] Yang A.B., Chen L.G., Xie Z.H., Sun F.R. (2019). Constructal operation cost minimization for in-line cylindrical pin-fin heat sinks. Int. J. Heat Mass Transf..

[51] Samal B., Barik A.K., Awad M.M. (2019). Thermo-fluid and entropy generation analysis of newly designed loops for constructal cooling of a square plate. Appl. Therm. Eng..

[52] Wang R., Xie Z.H., Yin Y., Chen L.G. (2020). Constructal design of elliptical cylinders with heat generating for entropy generation minimization. Entropy.

[53] Yang K., Huang W., Li X., Wang J.B. (2020). Analytical analysis of heat transfer and entropy generation in a tube filled with double-layer porous media. Entropy.

[54] Aminian E., Moghadasi H., Saffari H., Gheitaghy A.M. (2020). Investigation of forced convection enhancement and entropy generation of nanofluid flow through a corrugated minichannel filled with a porous media. Entropy.

[55] Bhattacharya A., Mahajan R.L. (2002). Finned metal foam heat sinks for electronics cooling in forced convection. J. Electron. Packag..

[56] Hung T.C., Huang Y.X., Yan W.M. (2013). Thermal performance of porous microchannel heat sink: Effects of enlarging channel outlet. Int. Commun. Heat Mass Transf..

[57] Hung T.C., Huang Y.X., Yan W.M. (2013). Thermal performance analysis of porous-microchannel heat sinks with different configuration designs. Int. Commun. Heat Mass Transf..

[58] Leng C., Wang X.D., Wang T.H., Yan W.M. (2015). Fluid flow and heat transfer in microchannel heat sink based on porous fin design concept. Int. Commun. Heat Mass Transf..

[59] Chen K.C., Wang C.C. (2015). Performance improvement of high power liquid-cooled heat sink via non-uniform metal foam arrangement. Appl. Therm. Eng..

[60] Gong L., Li Y.T., Bai Z., Xu M.H. (2018). Thermal performance of micro-channel heat sink with metallic porous/solid compound fin design. Appl. Therm. Eng..

[61] Li Y.T., Gong L., Xu M.H., Joshi Y. (2019). Hydraulic and thermal performances of metal foam and pin fin hybrid heat sink. Appl. Therm. Eng..

[62] Ghorbani M., Salimpour M.R., Vafai K. (2019). Microchannel thermal performance optimization utilizing porous layer configurations. Int. J. Heat Mass Transf..

[63] Rasam H., Roy P., Savoldi L., Ghahremanian S. (2020). Numerical assessment of heat transfer and entropy generation of a porous metal heat sink for electronic cooling applications. Energies.

[64] Wang T.H., Wu H.C., Meng J.H., Yan W.M. (2020). Optimization of a double-layered microchannel heat sink with semi-porous-ribs by multi-objective genetic algorithm. Int. J. Heat Mass Transf..

[65] Bejan A. (2004). Designed porous media: Maximal heat transfer density at decreasing length scales. Int. J. Heat Mass Transf..

[66] Ordonez J.C., Bejan A., Cherry R.S. (2003). Designed porous media: Optimally nonuniform flow structures connecting one point with more points. Int. J. Therm. Sci..

[67] Wildi-Tremblay P., Gosselin L. (2007). Layered porous media architecture for maximal cooling. Int. J. Heat Mass Transf..

[68] Lorente S. (2007). Constructal view of eclectrokinetic transfer through porous media. J. Phys. D Appl. Phys..

[69] Lorente S., Bejan A. (2006). Heterogeneous porous media as multiscale structures for maximum flow access. J. Appl. Phys..

[70] Azoumah Y., Neveu P., Mazet N. (2004). Constructal network for heat and mass transfer in a solid-gas reactive porous medium. Int. J. Heat Mass Transf..

[71] Azoumah Y., Neveu P., Mazet N. (2006). Constructal design combined with entropy generation minimization for solid-gas reactors. Int. J. Therm. Sci..

[72] Zhou S.B., Chen L.G., Sun F.R. (2007). Constructal entropy generation minimization for heat and mass transfer in a solid-gas reactor based on triangular element. J. Phys. D Appl. Phys..

